# Unilateral sagittal split osteotomy: effect on mandibular symmetry in the treatment of class III with laterognathia

**DOI:** 10.1186/s40510-020-00319-3

**Published:** 2020-07-06

**Authors:** Naji Abou Chebel, Maria Saadeh, Ramzi Haddad

**Affiliations:** 1 Division of Orthodontics and Dentofacial Orthopedics, Department of Otolaryngology Head and Neck Surgery, American University of Beirut Medical Center, Beirut, Lebanon; 2 Department of Oral and Maxillofacial Surgery, Faculty of Dental Medicine, Lebanese University, Beirut, Lebanon; 3 Department of Orthodontics, Faculty of Dental Medicine, Lebanese University, Beirut, Lebanon; 4 Department of Forensic Odontostomatology and Human Identification, Faculty of Dental Medicine, Lebanese University, Beirut, Lebanon

**Keywords:** Facial asymmetry, Unilateral sagittal split osteotomy

## Abstract

**Abstract:**

The bilateral sagittal split osteotomy is considered the standard surgery to correct facial asymmetries. More recently, unilateral sagittal split osteotomy (USSO) was used to treat such malocclusions.

**Aim:**

To assess facial symmetry following USSO in the treatment of class III laterognathia.

**Methods:**

Frontal facial photographs of four groups of patients were assessed: (1) pre-surgical group (*n* = 30) with skeletal asymmetry, (2) postsurgical group assessing patients of the first group 2 years after USSO, (3) control group (*n* = 30) of patients judged to have harmonious facial norms, and (4) mirrored group (*n* = 30) in which the control photographs were altered by duplicating the right half side of the face to replace the left half, thus creating perfectly symmetrical faces. All 120 photographs were distributed to 40 expert orthodontists to evaluate and score facial symmetry using the visual analog scale. Skin sensitivity and temporomandibular joint (TMJ) disorders were also assessed clinically pre and postsurgically.

**Results:**

Statistically significant differences were observed between the pre-surgical group and each of the postsurgical and control groups (*p* < 0.001). The control and postsurgical groups received similar scores of symmetry (*p* = 0.774). The mirrored group received statistically significantly higher symmetry scores than either of the control or the postsurgical groups (*p* < 0.001). A reduction in TMJ disorders was noted after USSO and all patients reported normal skin sensation 2 years post-surgery**.**

**Conclusion:**

When indicated, USSO is a dependable and practical surgical approach to correct facial asymmetries associated with class III malocclusion.

## Introduction

Despite the recognized preference for symmetry, lateral asymmetry is actually a normal phenomenon noted in shoe size, extremity length, cranial morphology, and also in the face [1–3]. Indeed, facial asymmetry is not uncommon in normal healthy individuals, reportedly accounting for 21 to 85% of dentoskeletal discrepancies [4] and particularly associated with the skeletal class III discrepancy [5–8]. The most notable outcome of hemi-mandibular asymmetry is often chin deviation from the facial midline [4].

The majority of facial asymmetries are minute and imperceptible, generally requiring no treatment [2, 3, 9]. Severe degrees of asymmetry often necessitate surgical intervention because of functional, psychological, and esthetic implications [10].

As many as 35% of patients presenting for orthognathic evaluation have been reported with lower facial third asymmetry [11]. The most widely employed mandibular orthognathic surgeries are the sagittal split ramus osteotomy and the intra-oral vertical ramus osteotomy [9]. Despite the popularity of the bilateral sagittal split ramus osteotomy (BSSRO) and its outcome reliability in symmetrical mandibular surgery (parallel advancement or setback) [12], its application to asymmetrical correction may result in an asymmetrical mandible despite the correction of chin position. When the right and left mandibular angles are symmetrical, any correction of laterognathia or cant may create interferences between the inner and outer tables of the sagittal splits and create asymmetry at the level of the ramus and angles [13]. To avoid bony interferences, Ellis proposed an additional cut to the inner table; however, when the mandibular angles are already asymmetrical, this additional procedure maintains the existing asymmetry. On the contrary, in such conditions, asymmetrical bony contact may displace the ramus laterally on one side and medially on the other side, potentially improving the symmetry at the level of the angles. Although desirable, these “yaw” movements are extremely difficult to accurately plan in advance because of the difficulty to have a reproducible reference for the surgical cuts and movements.

A potential solution to this problem is to correct mandibular asymmetry through a unilateral sagittal split osteotomy (USSO) when indicated. The unilateral cut allows the surgeon to use the opposite condyle as the center of rotation, thereby controlling the yaw of the mandible during surgery and achieving more predictable results. Despite early descriptions of the USSO [5], and subsequent case reports and case series [6, 7, 14, 15], controversy remains with respect to its effectiveness and complications. Our team has been conducting the USSO to correct class III laterognathia when mandibular translation could correct asymmetry with limited anteroposterior movement. The objective of this study was to evaluate the achieved facial symmetry after using the USSO technique in adult patients presenting with mandibular laterognathia, compared to a control group of adult patients without facial asymmetry.

## Material and methods

The study was approved by the Institutional Review Board of the American University of Beirut (#BIO-2017-0447). The surgical sample consisted of patients with class III laterognathia who did not present frontal occlusal canting. All patients underwent a USSO procedure coupled with a maxillary Le Fort I surgery. In 6 patients of the total sample, genioplasty was anticipated and performed in only the anteroposterior direction. The selection criteria included clinical and radiographic confirmation of mandibular asymmetry, and eligibility for the USSO upon clinical examination.

### Surgical planning and procedure

Eligibility for the USSO was determined through the “forced symmetry” concept, originally described by Grybauskas et al. for the BSSO [16]. The patients were instructed to gradually rotate the mandible to a centered position when a symmetrical lineup of the chin with the face was obtained. The patient was requested to maintain the mandible in that position, to ascertain visually and through palpation that the asymmetry at the level of the ramus and two gonial angles was visually corrected.

The surgical sequence included the mandible-first approach. An occlusal wafer was prepared and fabricated prior to surgery to facilitate sliding of the mandible to the centered position during surgery. After performing the sagittal split on one side, the mandible was moved using the opposite condyle as the center of rotation. The site of the split (right or left) was determined in the pre-surgical planning phase depending on the desired esthetic outcome with respect to mandibular advancement or setback to improve the facial profile. The USSO was performed on the same side of the deviation when the mandible required advancement and on the opposite side of the deviation for mandibular setback. Temporary maxillo-mandibular fixation was realized with the chin centered to the facial midline by rigid fixation of the unilateral sagittal split with three bicortical screws. Following mandibular fixation, a classical Le Fort I cut was performed, the maxilla advanced, then fixed to the mandible in ideal occlusion before finally applying rigid fixation with four L-shaped plates.

All surgical procedures were conducted under standard general anesthesia (with nasotracheal intubation) by the same surgeon, using an intra-oral approach. Intermaxilllary fixation was achieved with the use of inter-arch elastics in the direction of the surgery and was maintained for a period of 1 month. None of the patients experienced or reported any significant complications. Clinical follow-up was performed once a week for a period of 1 month. Postsurgical orthodontic treatment was completed within an average period of 6.5 months, in which inter-arch elastics were continued as necessary to optimize seating of the dental occlusion. All patients were recalled at a yearly basis for at least 2 years after finalizing surgical treatment.

### Study procedure

The photographs of two groups of patients, later stratified in 4 groups, formed the basis of the study (Table 1): (1) the surgical group: 30 adult patients who had received the USSO surgical procedure to correct their class III malocclusion with mandibular asymmetry and (2) the control group: 30 adult patients who consulted for reasons other than facial asymmetry, who were judged to be free of notable facial asymmetry. The faces of the control group were assessed clinically and on routine radiographs and photographs and were judged to be within acceptable norms, a procedure in concordance with previous studies comparing asymmetrical patients to control samples [17, 18].

**Table 1:** Selection criteria in the surgical and control groups

	Surgical group	Control group
Menton deviation assessed clinically and on frontal photographs	> 3 mm from the midsagittal plane/facial midline	< 1 mm from midsagittal plane/facial midline
Deviation of dental midlines	> 3 mm	< 1 mm
Skeletal relationships	Class III (ANB < 0°)	Class I (0° < ANB < 4°)

Frontal photographs were taken with a digital camera (500D, Macro lens canon EF 50 mm 1:2.5, Canon, Japan) with a white background. The distance between the white background and the camera was 1 m, which was a regular setup in the clinic for taking all orthodontic records, whereby practitioner and patient locations were marked with specific signs on the floor. The patients were instructed to stand with their heads in natural head position, while the practitioner judged the correctness of the position and adjusted it when needed. The patients were then instructed to look directly at the camera lens and maintain occlusal rest position. The camera settings (flash, aperture, and zoom) were standardized. Both ears were exposed, and the right and left distances between the exocanthus and the hairline were the same.

To form a sample of perfectly symmetrical faces, the photographs of all 30 control patients were modified and mirror imaged on Photoshop (Photoshop CS4 11.0.1, Windows 7) whereby the right half of the face was duplicated and flipped to build a perfectly symmetrical face.

**Fig. 1:**
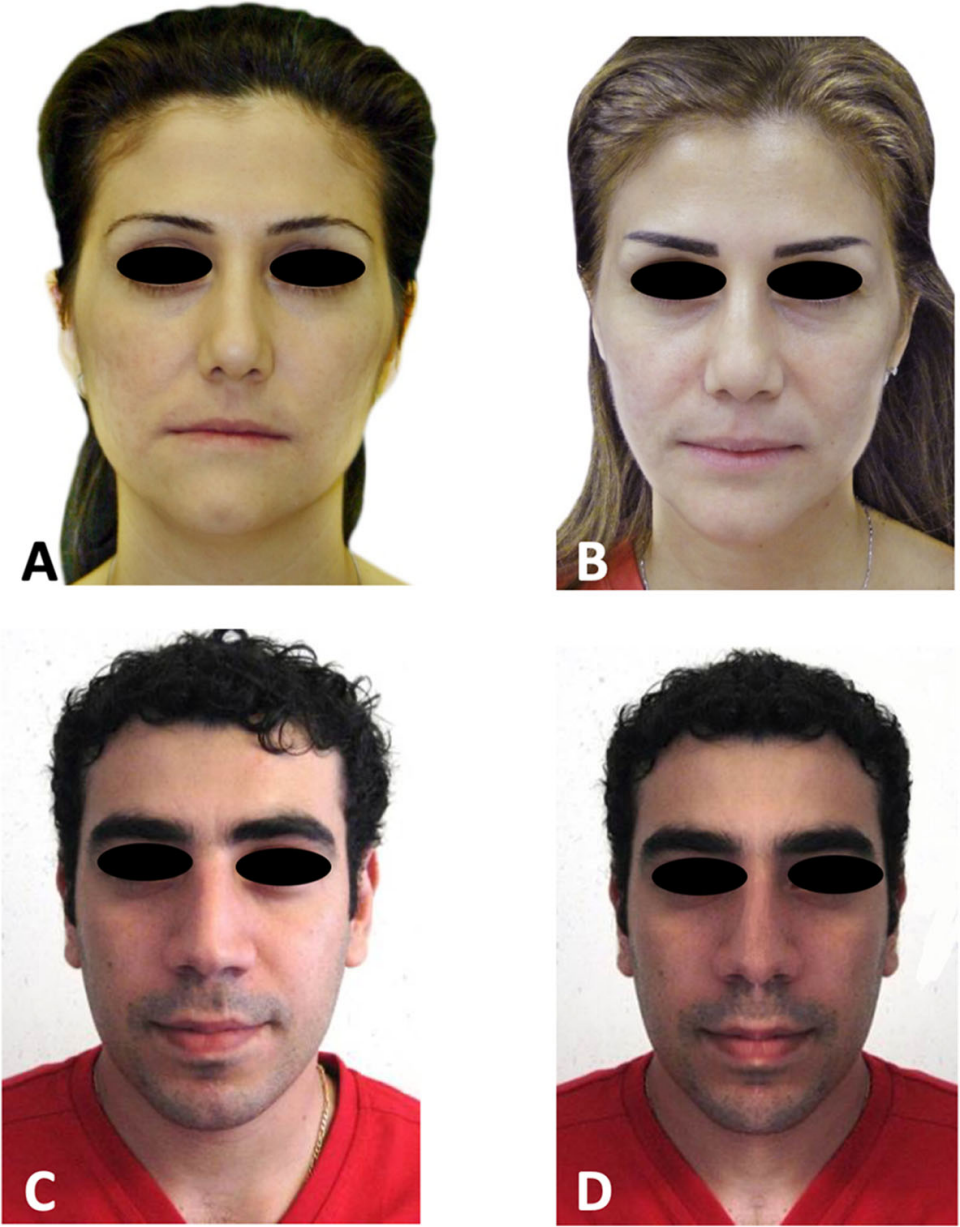
Frontal photographs of patients used for the panel study. **a** Pre-surgical photograph of patient with a facial skeletal asymmetry. **b** Postsurgical photograph of patient after USSO correction. **c** Photograph of patient judged to have well-balanced facial proportions. **d** Mirrored photograph of patient where the right half of the face is duplicated

The final study sample consisted of a total of 120 frontal extraoral photographs distributed into four groups (Fig. 1):

1. Pre-surgical group: pretreatment frontal photographs of the 30 patients in the surgical group (Fig. 1a),
2. Postsurgical group: 2 years postsurgical frontal photographs of the 30 patients in the surgical group (Fig. 1b),
3. Control group: original frontal photographs of the 30 patients in the control group (Fig. 1c),
4. Control mirrored group: mirrored photographs of the 30 patients in the control group (Fig. 1d).

**Fig. 2:**
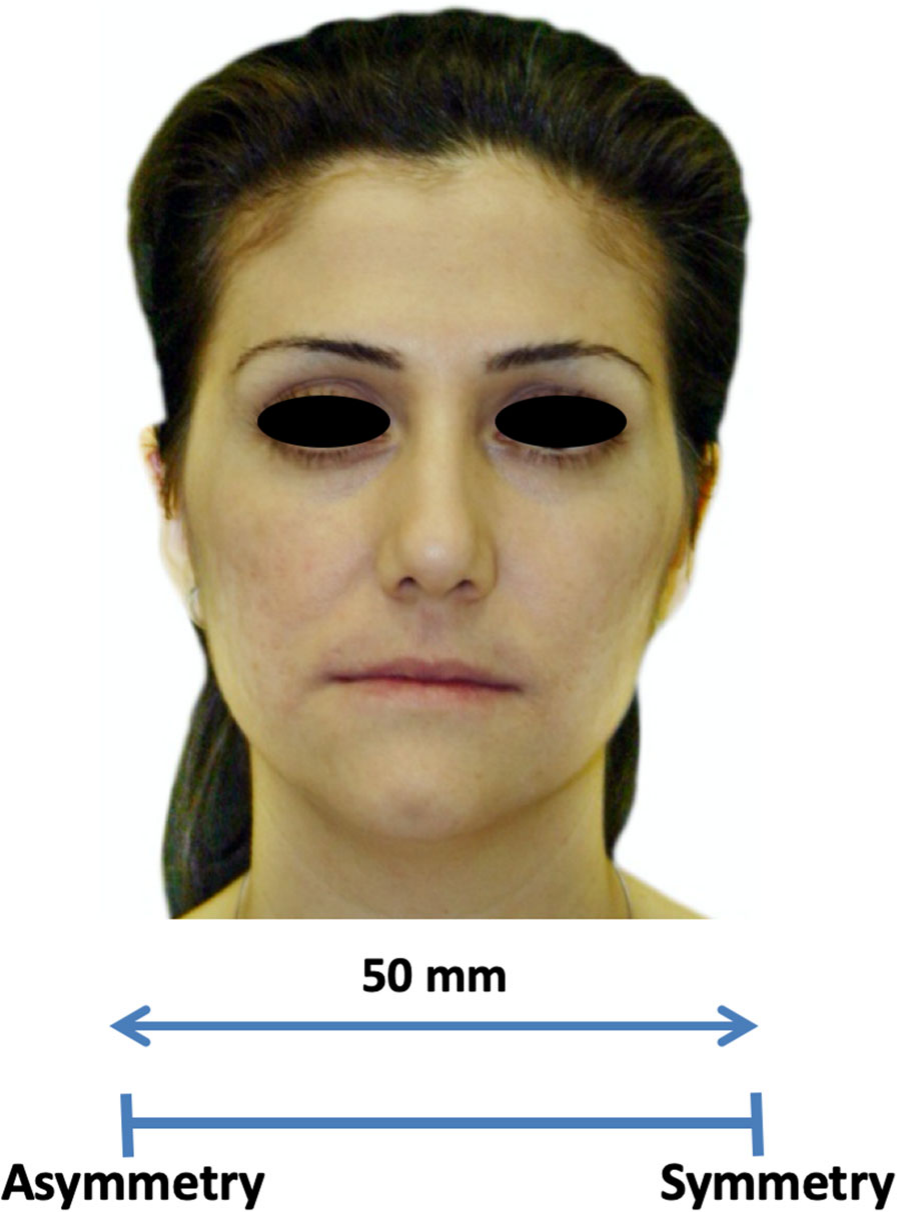
Visual analog scale (VAS) used to score facial symmetry ranging from “most asymmetric” (0 mm) on the left to “most symmetric” (50 mm) on the right

The colored photographs were numbered and printed individually on size A4 papers in random order, and the survey was distributed to a panel of 40 experienced orthodontists who had been practicing for at least 5 years. The orthodontists were requested to rate the facial symmetry on a 50-mm visual analog scale (VAS), ranging from “most asymmetric” (0 mm) on the left to “most symmetric” (50 mm) on the right; all assessments were subjective (Fig. 2). The VAS has been reported to have superior psychometric characteristics than discrete scales, and avoids the “ceiling effect” associated with discrete scales [19] and allows for the application of a wider range of statistical methods [20]. A single blind investigator measured the markings on the 50-mm VAS provided by each respondent for all 120 photographs using a digital caliper to the nearest 0.01 mm and recorded the measurement on a separate datasheet.

Facial skin sensitivity was tested clinically before treatment by asking all patients who underwent surgery to score on a scale ranging from 0 to 10 (0 being complete loss of sensitivity and 10 having normal sensation), the level of sensitivity loss. Temporomandibular joint (TMJ) disorders were also examined clinically by the same orthodontist. They included the assessment of the following: (1) pain at the level of both joints, (2) clicking, (3) mandibular deviation during opening, and (4) limitation of mouth opening. All findings were registered and the same criteria were tested 2 years postsurgically when the patients presented for the follow-up photographs. In addition, posteroanterior and lateral cephalograms, and panoramic radiographs were taken systematically on all patients before and after treatment in a digital radiographic machine (Instrumentarium Dental Company, Tuusula-Finland). The radiographs had a 1:1 ratio to actual size, and were processed through the manufacturer’s software (Cliniview, version 9.3.0.6). The condylar neck width and height were evaluated visually by superimposing the pre- and postsurgical radiographs for any anomaly in shape and interruption in outline.

A randomly selected sample of 10% of the total sample (*n* = 12, 3 from each group) was processed and measured by another examiner (MS) to determine the interexaminer reliability. In addition, the same investigator repeated 12 assessments of each group (at least 2 weeks after) to quantify the intra-examiner error.

### Statistical analysis

The repeated measures were evaluated with the two-way mixed effects intraclass correlations for absolute agreement on single measures to calculate both inter and intra-examiner errors. The 40 assessment scores provided by the panel of orthodontists were averaged for each photograph. Paired *t* tests were used to evaluate the difference between pre- and postsurgical group scores, in addition to the difference between control and their corresponding mirrored images. The difference between postsurgical and control groups and between postsurgical and mirrored groups was tested using the independent *t* test, after a Bonferroni correction for multiple testing. The significance was set at 0.05. Data were analyzed using the Statistical Package for Social Sciences (SPSS®, version 23.0, IBM®).

## Results

Reliability of repeated measurements was high for both intra and interexaminer errors. The intraclass correlation coefficients varied from 0.895 to 0.998 for the intra-rater and from 0.888 to 0.993 for the inter-rater reliability.

Following surgery, the surgical group was assigned a mean score of 28.81 ± 5.51, which was statistically significantly higher than the scores assigned to the pre-surgical photographs by a difference of 14.22 points (14.59 ± 6.4, *p* < 0.001, Table 2). The post-surgery mean score was nearly similar to the score assigned to the control group (29.26 ± 4.97, Table 3), the statistically non-significant difference averaging only 0.45 in favor of the control group. The mirror group, however, received the highest mean score of 42.67 ± 2.24, which was statistically significantly greater than both the postsurgical (*p* < 0.001, Table 3) and the control (*p* < 0.001, Table 2) groups, the difference being on average 13.86 and 13.41 points respectively.

**Table 2:** Comparison between control and mirrored groups and between pre-surgical and postsurgical groups

Group	Mean	SD	Mean difference	SE mean	Paired *t* test	
					*t*	*p* value
Control	29.26	4.97	− 13.41	5.12	− 13.854	< 0.001^*^
Mirrored	42.67	2.25				
Pre-surgical	14.59	6.41	− 14.22	5.52	− 14.344	< 0.001^*^
Postsurgical	28.81	5.51				

^*^Statistically significant, *p* < 0.001

**Table 3:** Comparison between the postsurgical group and both control and mirrored groups

Group	Mean	SD	Mean difference	SE mean	*t* test	
					*t*	*p* value
Control	29.26	4.97	0.45	1.37	0.328	0.744
Postsurgical	28.81	5.51				
Mirrored	42.67	2.25	13.86	1.07	12.883	< 0.001^*^
Postsurgical	28.81	5.51				

^*^Statistically significant, *p* < 0.001

All patients reported normal skin sensation 2 years postsurgical, with 38 patients scoring 10/10, and 2 submitting scores of 9/10.

Two patients reported bilateral pain at the level of the temporomandibular joint (TMJ) before surgery. The problems were diagnosed of neuromuscular origin. Palliative treatment instituted as other adjunct treatment (e.g., with nightguard) was not deemed to be advantageous because of the skeletal dysplasia. After surgery and at 2 years follow-up, no pain was registered on either side. Three other patients reported bilateral clicking before surgery; in only one of them, the clicking persisted after treatment but no additional therapy was recommended. Neither deviation nor limitation of mouth opening was detected.

The lack of condylar symptoms 2 years postsurgically was a positive indicator of condylar health. In addition, visual examination and superimposition of the pretreatment and the 2-year postsurgical panoramic radiographs did not reveal any apparent anomaly in shape or interruption in outline on the condylar surface and neck, nor in the width and height of the latter, discarding suspicious signs of condylar resorption necessitating further investigation.

**Fig. 3:**
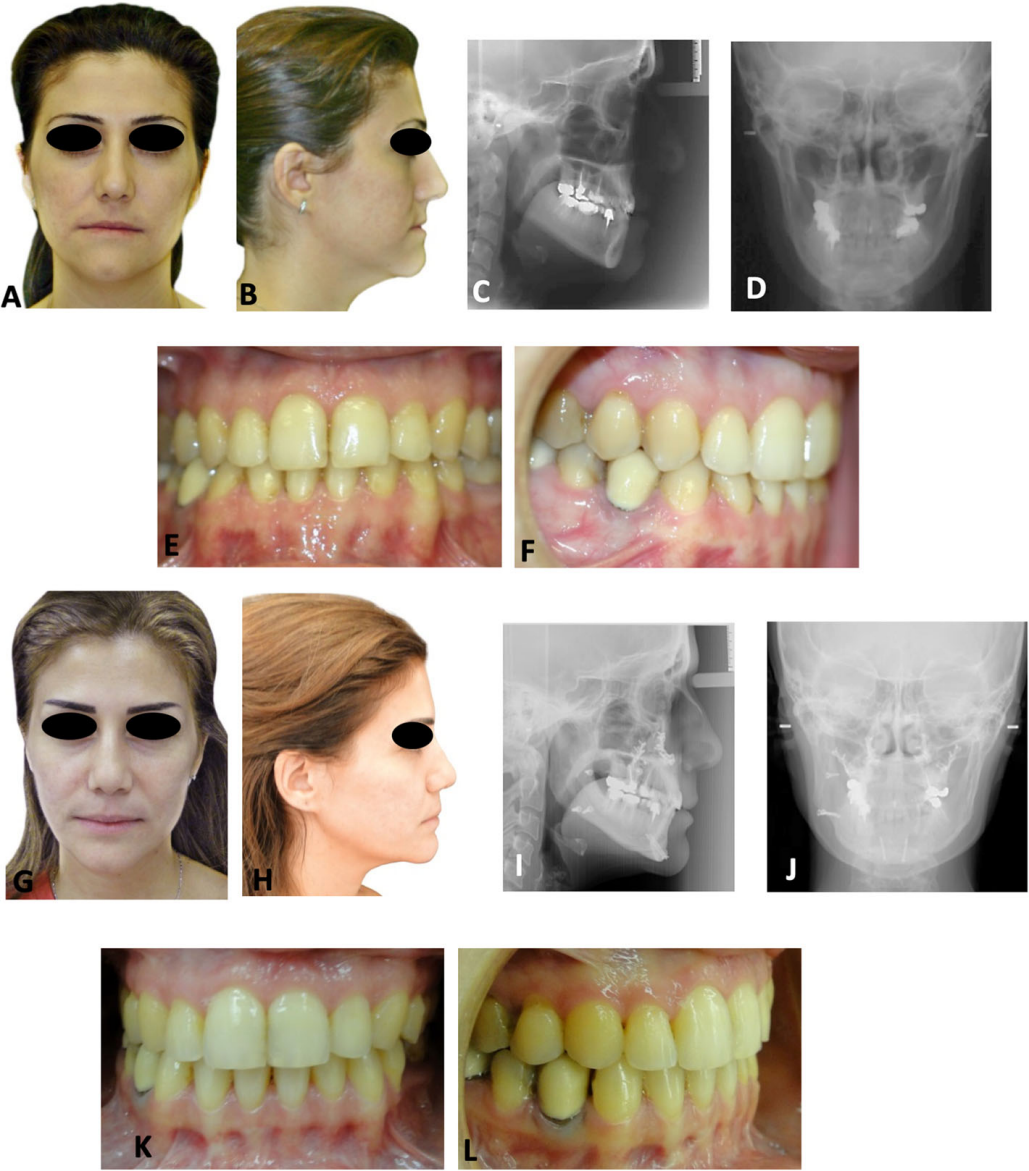
**a**, **b** Pretreatment facial and profile photographs of a 29-year-old female presenting with an orthodontically treated skeletal class III with facial asymmetry. **c** Lateral radiograph showing the dental camouflage of the class III malocclusion (ANB = − 3.5°) with severely retroclined mandibular incisors. **d** Posteroanterior radiograph displaying the skeletal asymmetry with an elongated left ramus causing deviation of the mandible to the right. **e**, **f** Intra-oral photographs reveal the outcome of the orthodontic camouflage. Note the deviation of the lower dental midline by 2 mm to the right. **g**–**l** Corresponding posttreatment facial, radiographic and occlusal records following the maxillary Le Fort I and USSO of the right mandibular ramus associated with genioplasty. Facial asymmetry was resolved (**g**, **j**), jaw relations rectified (ANB = 1.5°), and incisors in optimal inclinations over corresponding basal bones (**i**) and class I maintained (**k**, **l**). The midlines of the teeth, chin, and face were lined up

A representative patient report is shown in Fig. 3, highlighting the major orthodontic and surgical steps in the applied treatment protocol. The patient score for asymmetry was 9.76/50 before treatment and marked 39.42/50 after surgery.

## Discussion

Recent studies on mandibular structural morphology in subjects with menton deviation have revealed the existence of distinct mandibular phenotypes displaying asymmetrical lengths of the rami and varying vertical positions of the gonial angles [17, 20, 21]. Chen et al. observed that one of 3 identified mandibular subtypes that exhibited yaw rotation was least predictably corrected using bilateral sagittal split osteotomy, with residual ramus asymmetry persisting despite the correction of menton deviation [22]. In such conditions, the BSSRO would not produce reliable results because a proper reference in the mandible is difficult to find for treatment planning. The authors also noted that postsurgical bony contact between the two segments of the sagittal split differs on each side, potentially leading to unpredictable symmetry at the level of the rami and angles.

The etiology of a different phenotype may be related to functional or environmental factors. In a recent study assessing mandibular contour in patients with significant asymmetry, the mandibles actually displayed right-left symmetry and the optimal symmetry plane bisected the mandibular contour into two symmetrical halves [23]. The authors concluded that lower face asymmetry apparently results from mandibular deviation rather than structural mandibular asymmetry. They suggested that proper mandibular alignment should be the primary objective in the correction of facial asymmetry, an objective potentially easier attained with the USSO procedure in selected patients.

The inclusion of the mirrored faces as perfectly symmetrical faces helped delineate the accuracy of judging symmetry. The discrepancy between the mirrored faces score and that of the control and postsurgical groups is likely related to both the subjectivity involved in assessing facial symmetry particularly that the raters judged the photographs by inspection rather than using measurements, and the VAS property of inherently avoiding the ceiling effect. Moreover, perfectly symmetrical faces have been reported to be less attractive than natural faces with subtle asymmetries and are therefore unlikely to be considered as esthetically ideal [3, 24, 25].

A simple genioplasty by chin translation was not contemplated to correct the asymmetry for two reasons: the mandibular angles were asymmetrical; thus, the total asymmetry would not be corrected; the genioplasty would correct the chin midline but will not improve the asymmetry of the other parts of the mandible. Therefore, the patients who underwent a genioplasty in our sample were solely for the purpose of an anteroposterior correction, and no transversal translational movement was performed.

The eligibility for USSO through the “forced symmetry” concept was adopted for patient selection. The symmetry of the gonial angles after lineup of the chin with the face was an indication to apply the USSO technique. The patients included in our sample did not present frontal occlusal canting presurgically, which was an indicator that no vertical discrepancy would result from surgery and that any minor vertical discrepancy appearing during surgery would be corrected in situ. Such correction is facilitated by the sequence of mandible-first approach and the sagittal cut that allows the vertical movement between body and ramus.

During the pre-surgical simulation, the mandibular anteroposterior changes and the occlusal plane cant associated with the unilateral sagittal split were anticipated relative to the desired esthetic outcome, taking into consideration the anteroposterior mandibular surgical displacement (advancement or setback) needed to improve the facial profile. The correspondence between sagittal and transverse amounts of displacement followed the descriptions advanced by Beukes et al. [26] who performed virtual USSO procedures on randomly selected cone-beam scanned mandibles using a specialized 3D software. Accordingly, a 5-mm correction of mandibular asymmetry obtained at the dental midline when the operated side is advanced results in 3.17 mm advancement of the mandibular central incisors; mandibular setback causes 3.56 mm of posterior movement of the incisors. The anticipated anteroposterior movement is compensated by either maxillary advancement or setback depending on the pre-surgical esthetic planning. Patients in our sample did not require a transversal correction greater than 6 mm, thus reducing our maximal anteroposterior mandibular displacement to nearly 4 mm.

A salient question concerns rotation and remodeling of the condyle of the unoperated side, hence the adaptability of this joint. Both the anteroposterior and yaw movements of the mandible are determined by the center of rotation (on the unoperated condyle) and are nearly impossible to accurately simulate, thereby necessitating mandibular correction before maxillary advancement. In the study by Beukes et al., relatively small rotations of the unoperated condyle were reported in midline corrections of less than 5 mm [26]. In another study where the mandibular maximal movement was 7 mm anteroposteriorly, the condyle at the non-operated side rotated only 3–4° within the glenoid cavity, a rotation considered within the range of articular adaptation, ruling out subsequent functional dysfunctions [27]. To better determine the condylar rotational effect of the non-operated side, focused research employing CBCT images pre and post treatment (if allowed by IRB) should be conducted in relation to short and long-term stability of the results.

We derived our conclusion on stability from the fact that postsurgical results were similar to the control scores 2 years following surgery. In fact, postsurgical occlusion and condylar position are crucial to the maintenance of orthognathic surgery results [27]. In comparisons of occlusal parameters such as dental midline deviation, overbite, and overjet, as well as TMJ dysfunction following BSSRO or USSO in patients with lateral prognathism [28], no significant differences were found between treatment groups. The authors concluded that USSO was a stable alternative in patients with asymmetric mandibular prognathism, and reduced the operating time and morbidity when compared to BSSRO.

Our results on the absence of visible condylar root resorption on available panoramic radiographs (after surgery and at least 1 year post-surgery) are in agreement with findings by other authors. In comparison with published reports on USSO, Wohlwender et al. [29] did not find evidence of resorption or functional outcome when outlines of the condyles and the ascending ramus were digitized and traced on orthopantomographic (OPG) radiographs, and Beukes et al. [26] did not observe long-term symptoms.

In reports of patients treated with USSO, the patients did not exhibit TMJ dysfunction, pain, limited mouth opening following surgery, or evidence of condylar resorption [30, 31]. In the present study, similar results were found but the reported symptoms were not quantified for level of severity. Ideally, and in future research, a scoring system should be used to quantify the level of pain in the various categories of reported TMJ symptoms.

Although several reports on the use of mandibular USSO to correct facial asymmetries have been published [32–34], insufficient data are available to describe the technique and its outcome. Our study included 30 patients, but further studies are needed with larger samples that would also focus on the actual amounts of condylar rotation relative to mandibular movement (advancement or setback). Three dimensional imaging (CBCT) would be optimal to detect condylar position before and after surgery at the level of both joints, but the routine use of CBCT imaging, particularly as postsurgical follow-up, is not yet recommended in daily orthodontic practice because of radiation doses [35], and accordingly not allowed at many institutions (including ours). The stability of our surgical results and related TMJ dysfunction was followed up 2 years post-surgery which is a satisfactory time interval, but longer-term observation would be more conclusive.

## Conclusion

1. Unilateral sagittal split osteotomy in patients with class III malocclusion and mandibular asymmetry affecting the chin and rami/angles greatly improved facial symmetry; asymmetry was perceived after surgery at a level similar in control subjects. Underscoring this outcome is the fact that mirrored halves of the face that formed nearly perfectly symmetrical faces approached the absolute score on perfection.
2. The results were stable 2 years following surgery, reinforcing the proper indication for the USSO procedure and the application of “forced symmetry” as a screening tool to determine eligibility.
3. The key element in the planning and execution of the USSO method is the correction of the asymmetry at the level of the ramus and angles.
4. When indicated, the advantages of the USSO compared with the BSSO include reduced operating time and surgical trauma.

## Data Availability

The datasets used and/or analyzed during the current study are available from the corresponding author on reasonable request.

## Abbreviations

BSSRO: Bilateral sagittal split ramus osteotomy
USSO: Unilateral sagittal split osteotomy
CBCT: Cone beam computed tomography
CAD/CAM: Computer-aided design and computer-aided manufacturing
TMD: Temporomandibular disorder
TMJ: Temporomandibular joint
